# Marker-assisted enhancement of bacterial blight (*Xanthomonas oryzae* pv*. oryzae*) resistance in a salt-tolerant rice variety for sustaining rice production of tropical islands

**DOI:** 10.3389/fpls.2023.1221537

**Published:** 2023-09-25

**Authors:** Raj Kumar Gautam, Pankaj Kumar Singh, Krishnan Sakthivel, K. Venkatesan, Shyam S. Rao, M. Srikumar, Joshitha Vijayan, B. Rakesh, Soham Ray, Jameel Akhtar, Bharat Raj Meena, Sapna Langyan, Sharik Ali, S. L. Krishnamurthy

**Affiliations:** ^1^ Indian Council of Agricultural Research (ICAR)-Central Island Agricultural Research Institute, Port Blair, India; ^2^ Indian Council of Agricultural Research (ICAR)-National Bureau of Plant Genetic Resources, New Delhi, India; ^3^ Indian Council of Agricultural Research (ICAR)-Indian Institute of Oilseeds Research, Hyderabad, India; ^4^ Indian Council of Agricultural Research (ICAR)-National Bureau of Plant Genetic Resources (NBPGR), Regional Research Station, Thrissur, Kerala, India; ^5^ Indian Council of Agricultural Research (ICAR)-National Institute for Plant Biotechnology, New Delhi, India; ^6^ Indian Council of Agricultural Research (ICAR)-Indian Agricultural Research Institute, New Delhi, India; ^7^ Indian Council of Agricultural Research (ICAR)-Central Soil Salinity Research Institute, Karnal, India

**Keywords:** bacterial blight, marker-assisted backcross breeding, pyramiding, resistance, rice, salinity tolerance, tropical islands

## Abstract

**Introduction:**

Bacterial blight (BB) caused by Xanthomonas oryzae pv. oryzae is a major disease of rice, specially in the tropical regions of the world. Developing rice varieties with host resistance against the disease is the most effective and economical solution for managing the disease.

**Methods:**

Pyramiding resistance genes (Xa4, xa5, xa13,and Xa21) in popular rice varieties using marker-assisted backcross breeding (MABB) has been demonstrated as a cost-effective and sustainable approach for establishing durable BB resistance. Here, we report our successful efforts in introgressing four resistance genes (Xa4, xa5, xa13, and Xa21) from IRBB60 to CARI Dhan 5, a popular salt-tolerant variety developed from a somaclonal variant of Pokkali rice, through functional MABB.

**Results and discussion:**

Both BB and coastal salinity are among the major challenges for rice production in tropical island and coastal ecosystems. Plants with four, three, and two gene pyramids were generated, which displayed high levels of resistance to the BB pathogen at the BC3F2 stage. Under controlled salinity microplot environments, the line 131-2-175-1223 identified with the presence of three gene pyramid (Xa21+xa13+xa5) displayed notable resistance across locations and years as well as exhibited a salinity tolerance comparable to the recurrent parent, CARI Dhan 5. Among two BB gene combinations (Xa21+xa13), two lines, 17-1-69-334 and 46-3-95-659, demonstrated resistance across locations and years, as well as salt tolerance and grain production comparable to CARI Dhan 5. Besides salinity tolerance, five lines, 17-1-69-179, 46-3-95-655, 131-2-190-1197, 131-2-175-1209, and 131-2-175-1239, exhibited complete resistance to BB disease. Following multilocation testing, potential lines have been identified that can serve as a prospective candidate for producing varieties for the tropical Andaman and Nicobar Islands and other coastal locations, which are prone to BB and coastal salinity stresses.

## 1 Introduction

Rice (*Oryza sativa* L.) is the world’s primary carbohydrate source, feeding nearly half of the global population. India must produce 135–140 million tons of rice by 2030 to maintain self-sufficiency and meet future food grain demands (Gujja and Thiyagarajan, 2009). This goal must be achieved while considering the challenges posed by limited agricultural lands, depleting water supplies, declining soil productivity, emerging pests and pathogens, and the potential adverse effects of climate change. Historically, rice has also been the principal grain crop of the Andaman and Nicobar Islands, situated in the Bay of Bengal, despite its poor average yield. The farmers of the Andaman and Nicobar Islands belonging to diverse socio-ethnic groups and tribes mostly cultivate traditional rice varieties. The warm and humid conditions prevalent almost throughout the year in tropical islands facilitate the growth and multiplication of biotic stress factors, including diseases and insect pests. Among the biotic stresses, bacterial blight (BB) caused by *Xanthomonas oryzae* pv. *oryzae* is one of the most important diseases of rice, which spreads epidemically in susceptible cultivars and causes yield losses ranging from 20% to 50%, depending on weather conditions (Singh et al., 2011). The favorable temperature for the development of disease is between 25 and 34°C, with a relative humidity above 70%, which commonly coincides with the weather conditions of the islands. The problem is further exacerbated by susceptible varieties, poor management practices, and closed farming system scattered across different islands. Chemical disease management is not a recommended practice in these islands due to its high cost and negative environmental impact on terrestrial and marine ecosystems. The prevalence of BB has a visible and direct detrimental effect on the Paddy-cum-fish and duck-type farming systems, as well as an indirect impact on the coastal mangrove ecology (Chukwu et al., 2019). The most effective approach to minimizing losses is to develop resistant/tolerant rice varieties. Additionally, the Andaman and Nicobar Islands face challenges from coastal salinity and tsunami effects, which affect about one-third of the total area and are compounded by the region’s vulnerability to climate change. While salt stress poses a worldwide threat to agricultural production (Hopmans et al., 2021), the effects of salinity are exacerbated in high rainfall coastal and islands’ ecosystems due to the presence of acid sulfate soils (Sarangi et al., 2022).

The development of resistant cultivars carrying resistant genes has been the most effective and economical strategy for controlling BB disease without causing environmental pollution (Huang et al., 1997; Singh et al., 2001; Jena and Mackill, 2008; Sundaram et al., 2008; Rajpurohit et al., 2011; Dokku et al., 2013; Suh et al., 2013). To confer resistance against BB, more than 46 disease-resistance (R) genes have been identified and designated with a series from *Xa1* to *Xa46* (Fiyaz et al., 2022). Many of these genes are tagged with DNA-based markers, and some have been cloned and characterized (Kumar et al., 2020). The marker-assisted introgression of multiple resistance genes into a single genetic background is invariably preferred because the probability of simultaneous mutation at multiple loci to overcome the resistance is low (Koide et al., 2010).

Pokkali is a traditionally popular rice variety cultivated in the coastal saline areas of Kerala, India (Gregorio et al., 2002). It has been identified as a universal donor of salt tolerance genes, of which *Saltol* is the most prominent (Thomson et al., 2010). The analysis of phenotypic responses, genomic composition, and QTLs present in the salt-tolerant introgressed lines (ILs) developed from a salt-tolerant rice landrace “Pokkali” using SSR and SNP markers has revealed potential salt tolerance mechanisms in these ILs, including *Na^+^* dilution in leaves, vacuolar *Na^+^* compartmentalization, and possibly the synthesis of compatible solutes (De Leon et al., 2017). Besides salt tolerance, Pokkali has also been found to be nutritionally rich in various micronutrients, bran oil, and antioxidants such as oryzanols, tocopherols, and tocotrienols (Shylaraj et al., 2018). In a quest to develop an agronomically desirable plant type, induction of somaclonal variation in Pokkali led to the development of an elite variety called CARI Dhan 5 (Mandal et al., 1999), which is now an increasingly popular and successful salt-tolerant variety in Andaman and Nicobar Islands due to its wide seed dissemination and larger-scale adoption (Singh et al., 2020). However, it is important to note that CARI Dhan 5 is susceptible to BB, which remains a major disease of rice in the tropical Andaman Islands.

Through our systematic research, we have evaluated the relative efficacy of various resistance genes for managing BB disease, further complemented by analyzing the diversity, distribution, and virulence of the pathogen. This has provided a deeper understanding of the disease dynamics and has opened up new opportunities through the host pathogen approach under Bay Islands (Gautam et al., 2015; Gautam et al., 2016a; Gautam et al., 2016b; Sakthivel et al., 2016; Sakthivel et al., 2017; Sakthivel et al., 2021). Our previous study proved the effectiveness of four gene combinations (*Xa4, xa5, xa13*, and *Xa21*) carried by IRBB60 under Andaman Islands conditions (Gautam et al., 2013). This led us to pyramid these resistance genes into the popular salt-tolerant variety CARI Dhan 5 using marker-assisted backcross breeding, considering the effectiveness of these genes and the susceptibility of CARI Dhan 5 to BB.

## 2 Materials and methods

### 2.1 Plant materials and crossing scheme

**Figure 1:**
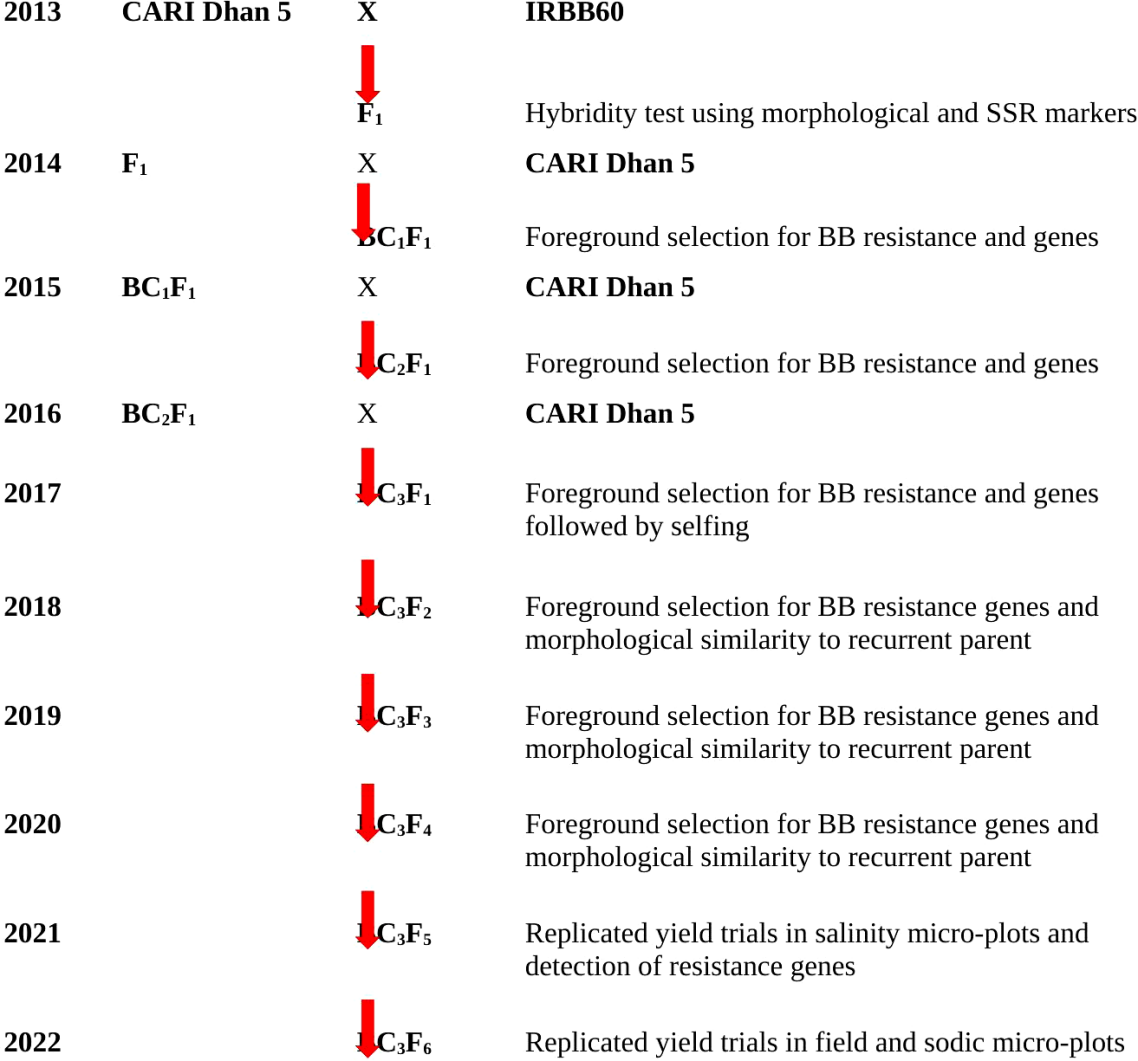
Back crossing scheme used to develop BB-resistant lines of CARI Dhan 5.

The donor parent, IRBB 60, possessed an impressive lineage of four BB resistance genes, namely, *Xa4*, *xa5*, *xa13*, and *Xa21*, which were incorporated into the background of IR24 through development at the International Rice Research Institute (IRRI), Philippines. On the other hand, the recurrent parent, salt-tolerant CARI Dhan5, was developed from the somaclonal variant selection from Pokkali (Mandal et al., 1999) and successfully notified for commercial cultivation in coastal saline and acid sulfate soils of the Andaman and Nicobar Islands. The crossing scheme for this hybridization was executed at Bloomsdale Research Farm, ICAR-CIARI, Port Blair, which is illustrated in **Figure 1**.

### 2.2 Bioassay against BB disease resistance

**Figure 2:**
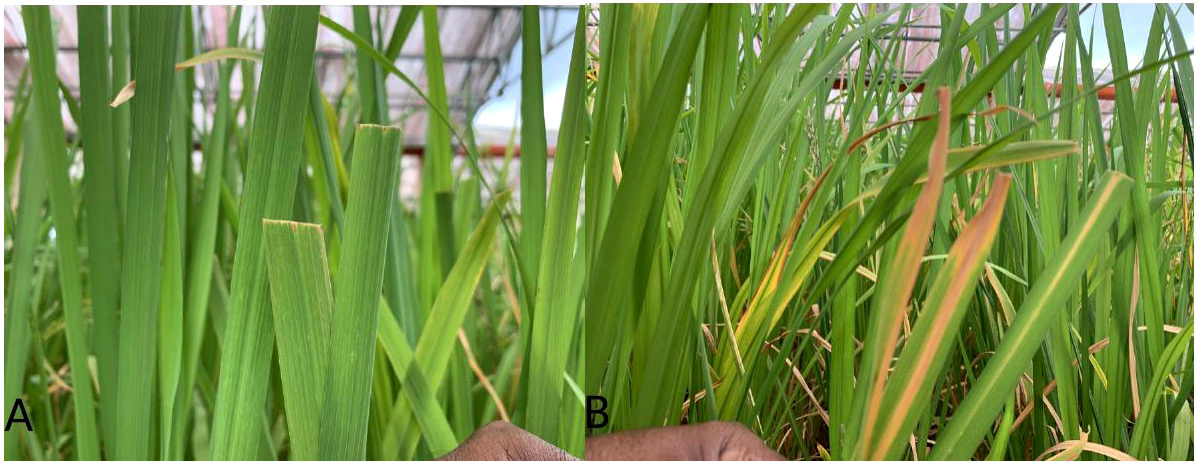
BB resistance testing in resistant introgression lines **(A)** and in the recurrent parent **(B)**.

Following 40 days of planting, both the parents and hybridization-derived plants were inoculated with a virulent isolate from the Andaman Islands using the clipping procedure as outlined by Kauffman (1973). After a period of 15 days post-inoculation, the lesion length of the inoculated leaves was meticulously measured. Disease scoring was carried out as per the standard evaluation system (SES, IRRI, 1996; Sakthivel et al., 2017). Based on the mean lesion length, the lines were categorized into four distinct groups, namely, resistant (<5 cm), moderately resistant (5–10 cm), moderately susceptible (10–15 cm), and susceptible (>15 cm). A BB-resistant and -susceptible reaction after inoculation is presented in **Figure 2**.

### 2.3 Evaluation of yield and other component traits of the pyramided lines

In the study, a total of 35 pyramided lines (BC_3_F_3/4_) and a single check (CARI Dhan5) were transplanted in both normal and saline environments in microplots and experimental fields located at ICAR-CIARI, Port Blair, in the Andaman and Nicobar Islands. Various agronomic traits were recorded, including plant height, days to 50% flowering, number of tillers per plant, panicle length, leaf length, leaf width, grain length, grain width, 100-seed weight, and phenotypically acceptability score (**Table S4**). To evaluate the distinctness, uniformity, and stability (DUS) of the 15 pyramided lines in normal soil, 41 agro-morphological traits were recorded at Bloomdale Farm of ICAR-CIARI, Port Blair, in the Andaman and Nicobar Islands, during the kharif season of 2020. The chosen advanced generation pyramided lines were phenotyped for their grain yield and related traits performance in both salinity stress (EC 5.0 dSm^−1^) and normal soil conditions in a controlled micro-plot facility at ICAR-CIARI, Port Blair, Andaman and Nicobar Islands. Here, it is pertinent to mention that the 30-day-old nursery seedlings were transplanted in microplots. After 7 days of seedling establishment, artificially prepared saline water (EC ~ 5.00 dS/m) was applied throughout the crop season to maintain this level of salt stress until maturity. On the other hand, only normal or fresh water (EC ~ 0.00 dS/m) was applied to the normal microplots. The pyramided lines were also screened for BB resistance along with their evaluation for grain yield and related traits.

### 2.4 DNA isolation and PCR amplification

The CTAB method described by Doyle and Doyle (1990) was used to isolate high-quality genomic DNA from rice. In brief, this method involves the use of CTAB, a cationic detergent, to extract DNA from the plant tissues, followed by precipitation and washing steps to purify the DNA. The resulting DNA was quantified and used as a template for PCR amplification. The PCR reaction mixture consisted of 50 ng of template DNA, 10 pmol of each of the primers, 200 μM of each dNTP, 1 × PCR buffer (10 mM Tris–HCl, pH 8.3, 50 mM KCl), 1.5 mM MgCl_2_, and 0.5 units of Taq DNA polymerase in a volume of 20 μl. The cycling conditions for the PCR reaction involved an initial denaturation step at 94°C for 5 min, followed by 35 cycles of amplification at 95°C for 30 s, 55°C for 30 s for primer annealing (Chen et al., 1997; Panaud et al., 1996), and 72°C for 1 min for extension. The final extension was carried out at 72°C for 5 min. Template DNA was followed with the following cycling conditions: 30 s primers annealing at 55°C. The PCR products and the DNA fragments produced were separated by gel electrophoresis using an agarose gel, and gel images were analyzed on an Alpha Imager (Alpha Innotech, USA).

### 2.5 Marker analysis

Sequence tagged site (STS) markers as well as functional PCR-based markers, Xa4F/Xa4R, for the *Xa4* gene (Ma et al., 1999; Sundaram et al., 2011); RG556 and xa5SF/xa5SR/xa5FM-RF/xa5FM-RR for the *xa5* gene (Hajira et al., 2016); RG136 and xa13-prom for *xa13* gene (Chu et al., 2006); and pTA248 for the *Xa21* gene (Song et al., 1995) were used for the foreground selection of resistance allele of each gene during each backcross generation (**Table 1**). In case of Xa4, the resistant allele is of size 150 bp while the susceptible allele is of size 120 bp. We have marked the identity of these bands in both the susceptible parent (SP) and the resistant parent (RP) to prevent any ambiguity. In case of the xa5 gene, the 424-bp band is common to all while a 313-bp band marks susceptible allele and a 134-bp band marks the resistant allele. We have marked the identity of these bands in both the susceptible parent (SP) and the resistant parent (RP) to prevent any ambiguity. The detection of *Xa13* and *Xa21* was performed as per Chu et al. (2006) and Song et al. (1995), respectively. Parental polymorphism survey was done between donor (IRBB60) and recipient (CARI Dhan 5) varieties using 200 HvSSR markers to select polymorphic markers associated with the recipient parents for background selection in later generations. The genomic contribution of the parent was determined by the assessment of SSR marker data in the selected recombinants. The marker-assisted backcross breeding approach was implemented from the F_1_ stage up to the BC_3_F_1_ generation. At each stage, foreground selection was carried out to identify plants carrying the BB resistance genes. Only progenies with the resistance alleles were advanced to the next generation (**Figure 1**).

**Table 1:** Microsatellite markers used between CARI Dhan 5 and IRBB60 for BB resistance genes in foreground selection under study.

BB resistance gene	Chromosome no.	Marker (s)	Marker distance	Primer sequences used for gene detection		Expected size (bp) for resistant homozygous	Reference
				Forward (5′-3′) Reverse (5′-3′)			
*Xa4*	11	Xa4F, Xa4R	0.5 cM	ATCGATCGATCTTCACGAGG	TGCTATAAAAGGCATTCGGG	150 bp	Ma et al., 1999; Sundaram et al., 2011
*xa5*	5	xa5SF, xa5SR xa5FM-RF xa5FM-RR		GTCTGGAATTTGCTCGCGTTCG AGCTCGCCATTCAAGTTCTTGAG	TGGTAAAGTAGATACCTTATCAAACTGGATGACTTGGTTCTCCAAGGCTT	424 bp, 134 bp	Hajira et al., 2016
*xa13*	8	xa13-prom F xa13-prom R		GGCCATGGCTCAGTGTTTAT	GAGCTCCAGCTCTCCAAATG	450 bp	Chu et al., 2006
*Xa21*	11	pTA248	0–1 cM	AGACGCGGAAGGGTGGTTCCCGGA	AGACGCGGTAATCGAAAGATGAAA	1,000 bp	Song et al., 1995

## 3 Data analysis

The experiments were performed in two to three replications and repeated twice for confirmation. The results were expressed as the mean ± SE of different independent replicates. An analysis of variance was performed in WASP software (version 2.0). *P* values of ≤0.05 were considered as statistically significant. The PCR amplification was performed in a Bio-Rad thermocycler (C1000 Thermal Cycler, Hong Kong, China). The images of the gel were analyzed on a Bio-Rad Gel Doc XR+System (California, USA).

## 4 Results

### 4.1 Pyramiding of BB resistance genes into CARI Dhan 5

The introgression of four BB resistance genes into CARI Dhan 5 was achieved via a crossing scheme (**Figure 1**) wherein IRBB60 (*Xa4*, *xa5*, *xa13*, and *Xa21*) was used as the donor parent. The results of the parental polymorphism survey revealed a significant level of polymorphism (23.5%) between CARI Dhan 5 (recurrent) and IRBB60 (non-recurrent) based on the analysis of 200 microsatellite markers. Among the markers surveyed, 47 were found to exhibit parental polymorphism. In addition, parental polymorphism was observed for all four target resistance genes, which was confirmed through the use of linked markers pTA248 (*Xa21*); RG136 (*xa13*); RG556, xa5SF/xa5SR (*xa5*); and Xa4F/Xa4R (*Xa4*).

In 2013–2014, F_1_ seeds were generated by crossing CARI Dhan 5 with IRBB 60. The BB-resistant F_1_ plants were confirmed based on hybridity for morphological and molecular markers of four resistance genes and were then pollinated by CARI Dhan 5 during the period 2014–2015 and 356 seeds of BC_1_F_1_ were obtained (**Table 2**). The progenies were thoroughly screened for BB resistance and the presence of linked genes through foreground selection, and BC_2_F_1_ crosses were attempted accordingly. In the Kharif season of 2015–2016, a total of 79 BC_2_F_1_ plants were screened using foreground selection, and only the plants exhibiting the presence of BB resistance genes were again pollinated by CARI Dhan 5 to obtain BC_3_ F_1_ seeds. In the year 2016–2017, a total of 3,505 BC_3_F_1_ plants were transplanted in the field, where foreground selection was carried out in conjunction with selection for CARI Dhan 5 phenotypes. Subsequently, the selected plants were self-pollinated to develop BC_3_F_2_ seeds.

**Table 2:** Number and nature of breeding lines developed in a cross CARI Dhan 5 x IRBB60 over different filial generations.

Year	Filial generation	No. of plants	BB-resistant individuals	Individuals having ≥ 3 BB resistance genes
2014	F_1_	10	10	10
2015	BC_1_F_1_	356	24	18
2016	BC_2_F_1_	79	18	9
2017	BC_3_F_1_	3,505	62	14
2018	BC_3_F_2_	60	28	8
2019	BC_3_F_3_	3,050	1,802	18
2020	BC_3_F_4_	2,600	1,640	20

### 4.2 Disease screening and marker genotyping of the BC_1_F_1_ population

To introgress BB resistance genes into CARI Dhan 5, single F_1_ plants were selected and further backcrossed with the recurrent parent. A total of 356 BC_1_F_1_ plants were produced and artificially inoculated with virulent *Xoo* isolates. Out of these, 24 plants scored highly for resistance to BB disease (**Table 2**). These twenty-four plants were subjected to foreground selection for the presence of BB resistance genes, namely, *Xa4*, *xa5*, *xa13*, and *Xa21*, by using corresponding linked markers. The molecular detection of the respective BB resistance genes, *Xa4*, *xa5*, *xa13*, and *Xa21*, in the BC_1_F_1_ plants was carried out, and specific bands were obtained as 19 (150 bp), 24 (300 bp), 16 (490 and 530 bp), and 18 (1,000 bp), respectively. Furthermore, the number of BC_1_F_1_ plants with different target resistance gene combinations obtained were as follows: (1) *xa5*, *xa13*, and *Xa21* (4 lines viz., 21, 54, 80, and 93); (2) *Xa4*, *xa5*, and *xa13* (1 line viz., 19); (3) *Xa4*, *xa5*, and *Xa21* (4 lines viz., 43, 53, 57, and 79); and (4) *Xa4*, *xa5*, *xa13*, and *Xa21*(9 lines viz., 15, 17, 46, 86, 88, 113, 118, 131, and 134). Out of the nine BC_1_F_1_ plants carrying all four target resistance genes, three plants (17, 46, and 131) exhibited the highest similarity with CARI Dhan 5 and were selected for further back-crossing with the recurrent parent to obtain BC_2_F_1_ seeds.

### 4.3 Disease screening and marker genotyping of the BC_2_F_1_ population

Out of 79 BC_2_F_1_ progenies produced, 18 plants showed a highly resistant reaction to bacterial leaf blight with a score of 1, while 14 plants showed resistant to moderate resistant reaction with a score of 3, as shown in **Table 2**. The 18 highly resistant plants were subjected to foreground selection for the presence of *Xa4*, *xa5*, *xa13*, and *Xa21* genes by using corresponding linked markers. In BC_2_F_1_ generation, the number of plants obtained with individual and different combinations of BB resistance genes were as [15 (*Xa4*), 17 (*xa5*), 12 (*xa13*), 4 (*Xa21*)] and [5 (*Xa4*, *xa5*, and *xa13*), 4 (*Xa21*, *xa13*, *xa5* and *Xa4*)] (**Figure S1**). Out of four BC_2_F_1_ plant progenies, three progenies exhibited the amplification of all four resistance genes, and therefore, BC_2_F_1_ plants were pollinated by CARI Dhan 5 to generate BC_3_F_1_ seeds.

### 4.4 Disease screening and marker genotyping of the BC_3_F_1_ population

A total of 3,505 BC_3_F_1_ backcross derivative progenies were produced by backcrossing the plants showing maximum recurrent genomes with the recipient parent, CARI Dhan5. Out of these, 62 plants showed high resistance to bacterial leaf blight with a score of 1 and were subjected to foreground selection for the presence of *Xa4*, *xa5*, *xa13*, and *Xa21* genes to identify plants that were homozygous for different resistant genes or their combinations using resistant gene linked markers (**Table 2**). In the case of individual R genes, 21 BC_3_F_1_ plants were positive for *Xa21*, 28 for *xa5*, 18 for *xa13*, and 21 for *Xa4*, whereas for different R gene combinations, the number of BC_3_F_1_ plants obtained for three and four gene combinations were as follows: three (lines 17-1-51, 17-1-55, and 46-5-143 with *Xa4*+*xa5*+*xa13* genes), two (lines 131-2-189 and 46-5-94 with *Xa4*+*xa13*+*Xa21* genes), two (lines 46-5-128 and 46-5-149 with *Xa4*+*xa5*+*Xa21* genes), one (line 131-2-182 with *xa5*+*xa13*+*Xa21* genes), and six (lines as 17-1-69, 131-2-175, 131-2-190, 46-3-95, 46-5-139, and 46-5-148 with *Xa21*+*xa13*+*xa5*+*Xa4* genes), respectively (**Supplementary Table 1**). The BC_3_F_1_ derivative progenies carrying all four resistance genes, i.e., 17-1-69, 131-2-175, 131-2-190, 46-3-95, 46-5-139, and 46-5-148, were selfed to obtain BC_3_F_2_ lines. Then, the line 46-5-148 was backcrossed to obtain 75 BC_4_F_1_ seeds with four resistance genes (*Xa4*, *xa5*, *xa13*, and *xa21*).

### 4.5 Genotyping and phenotyping of advanced breeding lines

**Figure 3:**
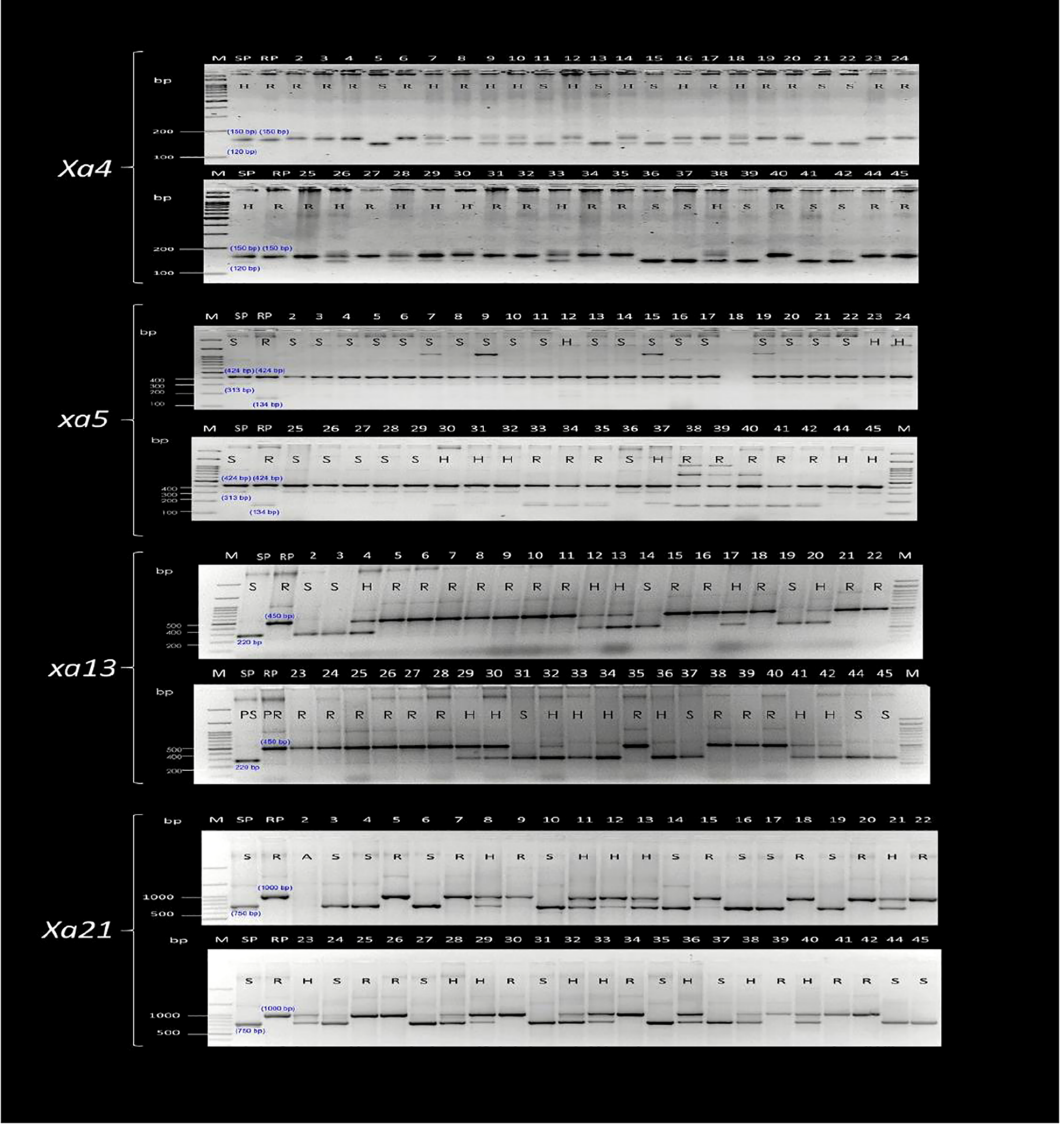
Marker profiling of the selected advanced breeding lines (ABLs) for foreground selection for *Xa21*, *xa13*, *xa5*, and *Xa4* genes. **M**, Marker lane; **SP/1**, CARI Dhan-5; **RP/43**, IRBB 60; **2**, 17-1-69-9; **3**, 17-1-69-34: **4**, 17-1-69-43; **5**, 17-1-69-55; **6**, 17-1-69-60; **7**, 17-1-69-72; **8**, 17-1-69-159; **9**, 17-1-69-179; **10**, 17-1-69-181; **11**, 17-1-69-204; **12**, 17-1-69-215; **13**, 17-1-69-316; **14**, 17-1-69-324; **15**, 17-1-69-334; **16**, 17-1-69-337; **17**, 17-1-69-346; **18**, 17-1-69-375; **19**, 17-1-69-384; **20**, 17-1-69-392; **21**, 46-3-95-640; **22**, 46-3-95-647; **23**, 46-3-95-648; **24**, 46-3-93-652; **25**, 46-3-95-655; **26**, 46-3-95-659; **27**, 46-3-95-683; **28**, 46-3-95-694; **29**, 46-3-95-917; **30**, 46-5-149-1185; **31**, 131-2-190-785; **32**, 131-2-190-795; **33**, 131-2-190-1190; **34**, 131-2-190-1196; **35**, 131-2-190-1197; **36**, 131-2-175-1207; **37**, 131-2-175-1205; **38**, 131-2-175-1209; **39**, 131-2-175-1223; **40**, 131-2-175-1224; **41**, 131-2-175-1208, **42**, 131-2-175-1239, **44**, AVT-CSTVT-2020-1; **45**, CSR 36.

**Figure 4:**
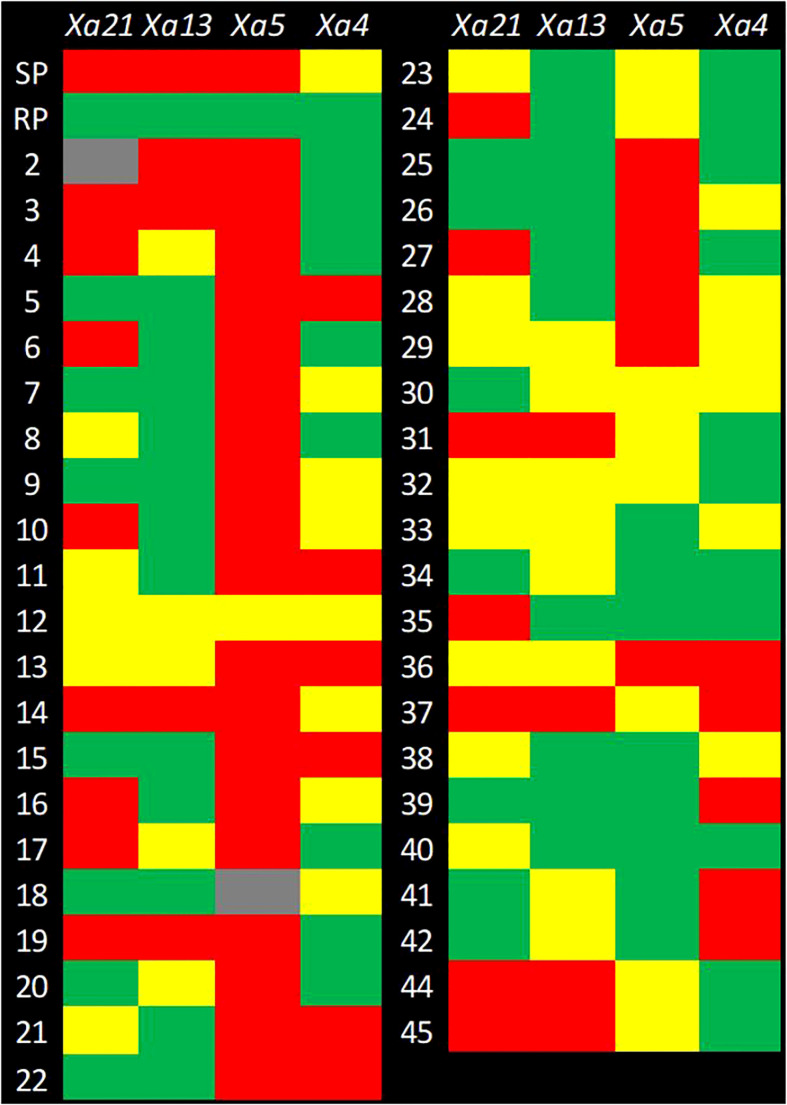
Genotyping of the selected advanced breeding lines (ABLs) for foreground selection. The checkerboard depicts the genotyping result for foreground selection. The rows represent genotypes and columns represent the genes for which genotyping has been performed. SP, Susceptible Parent; RP, Resistant Parent, 1–45^*^: Code number of SP/RP/selected ABLs for foreground selection. The Color Code: Green = Homozygous Resistant; Red = Homozygous Susceptible, Yellow = Heterozygous; and Gray = Missing Data. ^*^SP/1, CARI Dhan-5; **RP/43**, IRBB 60; **2**, 17-1-69-9; **3**, 17-1-69-34: **4**, 17-1-69-43; **5**, 17-1-69-55; **6**, 17-1-69-60; **7**, 17-1-69-72; **8**, 17-1-69-159; **9**, 17-1-69-179; **10**, 17-1-69-181; **11**, 17-1-69-204; **12**, 17-1-69-215; **13**, 17-1-69-316; **14**, 17-1-69-324; **15**, 17-1-69-334; **16**, 17-1-69-337; **17**, 17-1-69-346; **18**, 17-1-69-375; **19**, 17-1-69-384; **20**, 17-1-69-392; **21**, 46-3-95-640; **22**, 46-3-95-647; **23**, 46-3-95-648; **24**, 46-3-93-652; **25**, 46-3-95-655; **26**, 46-3-95-659; **27**, 46-3-95-683; **28**, 46-3-95-694; **29**, 46-3-95-917; **30**, 46-5-149-1185; **31**, 131-2-190-785; **32**, 131-2-190-795; **33**, 131-2-190-1190; **34**, 131-2-190-1196; **35**, 131-2-190-1197; **36**, 131-2-175-1207; **37**, 131-2-175-1205; **38**, 131-2-175-1209; **39**, 131-2-175-1223; **40**, 131-2-175-1224; **41**, 131-2-175-1208, **42**, 131-2-175-1239, **44**, AVT-CSTVT-2020-1; **45**, CSR 36.

Foreground selection was performed on 41 pyramided lines (BC_3_F_4_), as well as the parents (CARI Dhan 5 and IRBB 60) and a check (CSR 36), which were obtained by selfing lines 17-1-69, 131-2-175, 131-2-190, and 46-3-95. The selection was carried out using resistant gene linked markers to identify plants that were homozygous for different resistance genes or their combinations (**Figures 3**, **4**).

Thirty-five promising pyramided (BC_3_F_4_) lines were evaluated for yield-related parameters under two different conditions, viz., salinity and normal, under controlled micro-plot facility at ICAR-CIARI, Port Blair. Based on disease score and genotyping results, the lines containing bacterial leaf blight (BB) resistance genes along with other desirable characteristics of CARI Dhan 5 were identified (**Table S1**). Under saline conditions, line 17-1-69-334 showed a score of 1 (resistant) for BB disease, while lines 17-1-69-316, 17-1-69-324, 17-1-69-334, and 17-1-69-337 exhibited a score of 3 (moderately resistant). On the other hand, under normal conditions, the pyramided lines 17-1-69-375, 17-1-69-334, 17-1-69-215, 131-2-190-1196, and 131-2-175-1223 all scored 1, indicating resistance against BB disease (**Table S2**). The elite 17 pyramided lines selected based on genotyping and phenotyping of advanced breeding lines (ABLs) were further subjected to confirmation for BB resistance and salinity tolerance (Ece ~ 5.0 dSm^−1^) in micro-plots over the years (**Table 3**). Consequently, five lines, viz., 17-1-69-55, 17-1-69-72, 17-1-69-179, 17-1-69-334, and 17-1-69-375 [all carrying two BB resistance genes (*Xa21* and *xa13*)], were found to retain the original CARI Dhan 5 salinity tolerance with a salinity score of 1 (**Table S2**). A total of seven derivatives, viz., 17-1-69-55, 17-1-69-179, 17-1-69-334, 46-3-95-659 (*Xa21* and *xa13*), 131-2-190-1196 (*Xa21*, *xa5*, and *Xa4*), 131-2-190-1197 (x*a13*, *xa5*, and *Xa4*), and 131-2-175-1223 (*Xa21*, *xa13*, and *xa5*), were found to have complete resistance as shown in **Table S3**. Interestingly, five lines, viz., 17-1-69-179 (*Xa21* and *xa13*), 46-3-95-655 (*Xa21*, *xa13* and *Xa4*), 131-2-190-1197 (*xa13*, *xa5*, and *Xa4*), 131-2-175-1209 (*xa13* and *xa5*), and 131-2-175-1239 (*Xa21* and *xa5*), were identified to possess desirable dual attributes of salinity tolerance and BB resistance, as shown in **Table S2**. Yield and yield-related traits of the above-mentioned pyramided lines at the micro-plot as well as field levels are presented in **Table S2**. In the saline micro-plots (EC 5.0 dSm^−1^), five lines viz., 131-2-175-1239 (146.9), 17-1-69-72 (145.5), 17-1-69-375 (131.8), 46-3-95-647 (130.6), and 46-3-95-659 (124.6) demonstrated grain yield (expressed in g per 20 plants) greater than or equal to that of CARI Dhan 5 (123.4). Meanwhile, under normal soil conditions, lines, viz., 17-1-69-334 (300.2), 17-1-69-179 (272.9), 46-3-95-655 (265.1), 17-1-69-375 (232.8), and 46-3-95-647 (232.8), exhibited higher grain yield than CARI Dhan 5 (187.6). The lines 46-3-95-659 (6,183.1), 46-3-95-647 (5,997.9), 17-1-69-375 (5,838.5), 131-2-175-1209 (4,629.6), and 131-2-175-1223 (4,526.8) demonstrated grain yield (kg ha^−1^) above or comparable to that of CARI Dhan 5 (4434.2) under natural field-level conditions (**Table S3**). It is also noteworthy to mention that the 17 pyramided BC_3_F_3_ lines were also subjected to DUS characterization using 41 rice-descriptor traits. The results showed that the BB-resistant and salinity-tolerant derivatives exhibited agro-morphological similarity with the recurrent parent, CARI Dhan 5, indicating the retention of the host background in these agronomically targeted lines. The details of this analysis are presented in **Table S4**. Here, lines 17-1-69-375 and 46-3-95-647 performed well for the grain quality and yield-traits.

**Table 3:** Bacterial blight disease reaction of parental and pyramided lines against *Xoo* strains over the years and locations along with salinity score.

Parental and pyramided (BC_3_F_3_ and BC_3_F_4_) lines	Presence of BB gene combination in the parents/pyramided lines	Bacterial blight (BB) disease score						Salinity score	
		Field level				Micro-plot		Micro-plot	
		Port Blair			New Delhi	Port Blair		Port Blair	
		2020	2021	2022	2022	2021	2022	2021	2022
CARI Dhan 5	*-*	7	5	5	7	7	5	1	1
17-1-69-55	*Xa21+ xa13*	1	1	1	1	1	1	1	1
17-1-69-72	*Xa21+ xa13*	1	5	1	5	5	1	1	1
17-1-69-179	*Xa21+ xa13*	3	1	1	1	1	1	3	1
17-1-69-334	*Xa21+ xa13*	1	1	1	1	1	1	1	1
17-1-69-375	*Xa21+ xa13*	3	5	1	5	5	1	1	1
46-3-95-647	*Xa21+ xa13*	3	5	3	5	5	3	5	1
46-3-95-655	*Xa21+ xa13+ Xa4*	1	3	1	3	3	1	5	1
46-3-95-659	*Xa21+ xa13*	1	1	1	1	1	1	3	3
131-2-175-1196	*Xa21+ xa5+ Xa4*	1	1	1	1	1	1	1	3
131-2-175-1197	*xa13+ xa5+ Xa4*	3	1	1	1	1	1	3	1
131-2-175-1209	*xa13+ xa5*	1	3	3	3	3	3	3	3
131-2-175-1223	*Xa21+ xa13+ xa5*	1	1	1	1	1	1	3	1
131-2-175-1224	*xa13+ xa5+ Xa4*	1	5	1	5	5	1	3	3
131-2-175-1208	*Xa21+ xa5*	3	3	5	5	3	5	5	3
131-2-175-1239	*Xa21+ xa5*	3	1	3	1	3	3	3	3
IRBB 60	*Xa21+ xa13+ xa5+ Xa4*	1	1	1	1	1	1	9	5

## 5 Discussion

Salinity is a major abiotic stress for rice crops in coastal regions and the situation is expected to worsen due to climate change and the increasing number of irrigation networks. CARI Dhan 5, a somaclonal variant of the legendary rice landrace Pokkali, is a widely cultivated rice variety in the Andaman and Nicobar Islands due to its high yield and remarkable tolerance to coastal saline and acid sulfate soils (Mandal et al., 1999; Singh et al., 2020). However, CARI Dhan 5 is susceptible to BB disease caused by *X. oryzae* pv. *oryzae*, which is one of the most devastating diseases of rice. Being a tropical island, the hot and humid climate of the Andaman and Nicobar Islands provides congenial conditions for the completion of the disease triangle. In our previous studies, we conducted virulence profiling of *Xoo* isolates from the Andaman Islands, which revealed the prevalence of seven pathotypes of *X. oryzae* pv. *oryzae* belonging to two different clonal complexes (Sakthivel et al., 2021). Among these, pathotypes VI and VII were found to be highly virulent. Our analysis using multilocus sequence typing based on nucleotide sequence polymorphism in nine housekeeping genes, *dnaK*, *fyuA*, *gyrB* (two loci), *rpoD*, *fusA*, *gapA*, *gltA*, and *lepA*, further revealed that *Xoo* strains infecting rice in the Andaman and Nicobar Islands comprise a mixture of isolates representing mainland India and the islands, which might be due to trans-boundary movement of the BB pathogen through rice seeds (Sakthivel et al., 2021).

A combination of *Xa4*+*xa5*+*xa13*+*Xa21* has been found to confer broad-spectrum resistance to BB disease caused by different strains of *Xoo* reported from different parts of the world. Moreover, studies conducted over the years and in multiple locations have demonstrated that the resistance imparted by this gene combination does not depict any sign of breakdown, making it a promising approach to combating the disease (Shanti and Shenoy, 2005; Pradhan et al., 2015). Therefore, in this study, we aimed to introgress these four BB resistance genes (*Xa21*, *xa13*, *xa5*, and *Xa4*) into the genetic background of CARI Dhan 5, a coastal salinity-tolerant rice cultivar, using marker-assisted breeding.

The genetic background of the recipient parent is known to play a significant role in the successful integration and expression of gene pyramids. In this context, our previous research on five distinct genetic backgrounds carrying *xa5*+*xa13*+*Xa21* combinations revealed that the rice lines with IMP ASD16/60 and Improved Samba Mahsuri imparted complete resistance against all pathotypes of Andaman (Sakthivel et al., 2017). Fortunately, there are several resistance genes that confer resistance against *Xoo*. Notably, *Xa21*, *xa13*, and *xa5* have been reported to impart strong levels of resistance against *Xoo* strains prevalent in India (Goel et al., 1998; Shanti et al., 2001; Joseph et al., 2004). *Xa21* and *Xa4* are well-studied dominant resistance genes encoding receptor-like kinases (RLKs) (Song et al., 1995; Leach et al., 2001), while the *Xa21* protein contains a nucleotide-binding site and leucine-rich repeat (*NBS-LRR*) domain (Song et al., 1995) and *Xa4* protein belongs to the category of wall-associated kinases (WAK) family (Leach et al., 2001). These two genes exhibit complete dominance, function independently, and are cumulative in nature, indicating their crucial role in two distinct pathways of resistance (Li et al., 2001). On the other hand, the *xa13* gene is a recessive resistance allele of the susceptibility gene Os-*8N3* (the rice homolog of the nodulin *MtN3*) (Chu et al., 2006; Antony et al., 2010). The allele *xa13* contains a mutation in the promoter region that inhibits binding of the transcription factor and subsequent expression of the susceptibility gene (Antony et al., 2010). Similar to *xa13*, *xa5* is also a recessive resistance gene that encodes a variant form of the basal transcription factor TFIIAγ. This variant form, resulting from a missense mutation, cannot bind to the promoter region of the susceptibility locus, providing resistance to Xoo (Han et al., 2020). Together, the two dominant resistance genes *Xa4* and *Xa21* confer vertical resistance, making the plant nearly immune, while the two recessive genes *xa13* and *xa5* offer strong horizontal resistance, which is durable in nature.

Previous studies have successfully generated four- or three-gene pyramids in popular rice cultivars including *Mahsuri* (*Xa4+xa5+xa13+Xa21*) (Shanti et al., 2010); *Tapaswini* (*Xa4+xa5+xa13+Xa21*) (Dokku et al., 2013); *Swarna* (*Xa4+xa5+xa13+Xa21*) (Pradhan et al., 2019); *Putra-1* (*Xa4+xa5+xa13+Xa21*) (Chukwu et al., 2020); *Ranidhan* (*Xa4+xa5+xa13+Xa21*) (Pradhan et al., 2022); *Basmati-385* (*Xa4+xa5+xa13+Xa21*) (Ullah et al., 2023); *PR106* (*xa5+xa13+Xa21*) (Singh et al., 2001); *Jalmagna* (*xa5+xa13+Xa21*) (Pradhan et al., 2015); *Samba Mahsuri* (*xa5+xa13+Xa21*) (Sundaram et al., 2008); *ASD 16* (*xa5+xa13+Xa21*) (Ramalingam et al., 2020); and *ADT 43* (*xa5+xa13+Xa21*) (Ramalingam et al., 2020). Recent reports and reviews further suggest that the use of *Xa4* might be redundant and that the gene combination of *xa5+xa13+Xa21* is sufficient for providing resistance, which has been extensively used in marker-assisted backcross breeding in rice (Gautam et al., 2015; Fiyaz et al, 2022). It is worth noting that more than 70 rice varieties or hybrid parental lines have been improved for their BB resistance alone or in combination with genes/*QTL*s conferring tolerance to other biotic and abiotic stresses by the team.

In our previous study, we conducted a screening of 21 IRBB differentials that possessed individual *Xa1* to *Xa21* gene(s) as well as different gene combinations (Gautam et al., 2015). Based on these findings, it is concluded that three individual genes, namely, *Xa4*, *Xa7*, and *Xa21*, along with four combinations, *viz. Xa4+xa5* (IRBB50), *Xa4+Xa21* (IRBB52), *xa5+xa13+Xa21* (IRBB59), and *Xa4+xa5+xa13+Xa21* (IRBB60), conferred resistance against all the tested isolates from the Andaman Islands. IRBB60, in particular, has been a successful donor in rice breeding programs for conditioning BB resistance, as reported in several studies (Shanti et al., 2010; Dokku et al., 2013; Chukwu et al., 2019; Pradhan et al., 2019). Therefore, we employed a breeding strategy using IRBB60 as the donor parent and CARI Dhan 5 as the salt-tolerant recurrent parent to introgress at least three target *Xa/xa* genes into CARI Dhan 5. As a result, we successfully obtained a total of 14 BC_3_F_1_ plants with the desired gene combination. According to our findings, six BC_3_F_1_ progenies (17-1-69, 131-2-175, 131-2-190, 46-3-95, 46-5-139, and 46-5-148) were identified to harbor four gene pyramids (*Xa21*+*xa13*+*xa5*+*Xa4*). Additionally, three lines (17-1-51, 17-1-55, and 46-5-143) possessed *Xa4*+*xa5*+*xa13* genes, while two lines (131-2-189 and 46-5-94) contained *Xa4*+*xa13*+*Xa21* genes. The gene combination *Xa4+xa5+Xa21* was identified in two lines (46-5-128 and 46-5-149), while the derivative 131-2-182 possessed the *xa5+xa13+Xa21* combination. Therefore, these pyramided lines hold great promise as potential candidates for developing BB-resistant versions of CARI Dhan 5 for commercial release and cultivation in the coastal saline and acid sulfate soils of the Andaman and Nicobar Islands. In this study, we identified five resistant derivatives having three gene combinations, viz, 131-2-175-1223 (*xa5*+*xa13*+*Xa21*), 46-3-95-655 (*Xa21*+*xa13*+*Xa4*), 131-2-190-1196 (*Xa21*+*xa5*+*Xa4*), 131-2-190-1197 (*xa13*+*xa5*+*Xa4*), and 131-2-175-1224 (*xa13*+*xa5*+*Xa4*). Similar to 131-2-175-1223, the *xa5+xa13+Xa21* combination has been proven to confer durable and broad-spectrum resistance in many popular rice varieties in India (Singh et al., 2001; Sundaram et al., 2008; Pradhan et al., 2015; Ramalingam et al., 2020). The disease reaction scoring over the years and locations established the resistance of the progenies in the following order: 131-2-175-1223 (*xa5*+*xa13*+*Xa21*) > 17-1-69-55, 17-1-69-72, 17-1-69-179, 17-1-69-334, 17-1-69-375, 46-3-95-647, 46-3-95-659 (*xa13*+*Xa21*) > 131-2-175-1208, 131-2-175-1239 (*xa5*+*Xa21*) > 131-2-175-1209 (*xa5*+*xa13*). It is evident from our gene-pyramided lines that an enhanced level of BB resistance is conditioned when the *Xa21* gene (dominant allele) complements an allele of the *xa5* or *xa13* or both (recessive alleles). This finding is in line with several other earlier observations (Sanchez et al., 2000; Singh et al., 2001; Ramalingam et al., 2017) and is probably achieved due to the sword-shield effect mediated by resistance gene expression (the sword) coupled with non-expression of susceptibility gene (the shield). Four pyramided lines showed the presence of the *Xa4* gene with other genes (*Xa21*/*xa13*/*xa5*) in combination as 46-3-95-655 (*Xa21*+*xa13*+*Xa4*), 131-2-190-1196 (*Xa21*+*xa5*+*Xa4*), 131-2-190-1197 (*xa13*+*xa5*+*Xa4*), and 131-2-175-1224 (*xa13*+*xa5*+*Xa4*) with a BB disease reaction score of 1 or 3 over the years and locations. We also observed that though the recurrent susceptible parent CARI Dhan 5 is heterozygous for the *Xa4* gene, *Xa4* gene is reported as a weak gene imparting incomplete resistance against very few isolates in India (Mishra et al., 2013). Incomplete resistance of *Xa4* was also reported by our group in an earlier study under Andaman and Nicobar Island conditions (Gautam et al., 2015). Therefore, although CARI Dhan 5 harbors the *Xa4* allele, it behaves as a susceptible genotype to the *Xoo* races prevalent in these islands. Hence, it is not unusual for the susceptible parent CARI Dhan 5 to contain both resistant alleles (150 bp) and susceptible alleles (120 bp) and possess heterozygous genetic constitution for the said locus.

The DUS characterization of pyramided lines for 41 agro-morphological and descriptor traits confirmed that the advanced elite lines recovered the genetic background of CARI Dhan 5 in addition to BB resistance (refer to **Table S6** for details). It was imperative to confirm the retention of salinity tolerance in the ILs as compared to CARI Dhan 5. Based on the phenotypic scoring method, we found the ILs, viz., 17-1-69-55, 17-1-69-179, 17-1-69-334,131-2-175-1196, and 131-2-175-1197, as BB resistant (disease score 1) as well as salt tolerant (salinity score 1). Further evaluation under the saline micro-plot facility (EC 5.0 dSm^−1^) revealed that the pyramided line, viz., 17-1-69-334, exhibited a consistent score of 1 (resistant/tolerant) for both BB and salinity stresses across years and locations, with comparable grain yield performance of CARI Dhan 5. Overall, the results indicate that the salt-tolerant lines, viz., 17-1-69-179, 46-3-95-655, 131-2-190-1197, 131-2-175-1209, and 131-2-175-1239, also demonstrated complete resistance to BB disease. In view of their potential for dual attributes, these promising derivatives are being nominated for multi-location yield trials, under the All-India Co-ordinated Rice Improvement Program with their potential for release as BB-resistant and salt-tolerant varieties for cultivation in BB-prone and coastal saline conditions.

## 6 Conclusion

High salt concentrations in soils can cause a significant decline in rice yield and quality, and in some cases, it may even lead to complete crop failure (Xiao et al., 2020). CARI Dhan 5, a somaclonal variation-derived semi-dwarf variety from salt-tolerant rice landrace Pokkali in ICAR-CIARI, Port Blair, is a preferred rice variety in Andaman and Nicobar Islands due to its high yield, favorable grain quality, and high salinity tolerance. However, it is susceptible to BB disease, which is prevalent in the island’s environmental conditions. The present study endeavored to use marker-assisted backcross breeding to pyramid four BB resistance genes (*Xa4*, *xa5*, *xa13*, and *Xa21*) in the background of CARI Dhan 5. Consequently, five stabilized rice lines combining salt tolerance with BB resistance have been developed in the genetic background of CARI Dhan 5. The development of these improved CARI Dhan 5 lines is expected to have a strong and favorable impact on eco-friendly rice cultivation in the geographically remote Andaman and Nicobar Islands, which are affected by coastal salinity and BB disease.

## Data availability statement

The original contributions presented in the study are included in the article/**Supplementary Material**. Further inquiries can be directed to the corresponding authors.

## Author contributions

RG: Conceptualization, methodology, project execution, administration, primary draft, and editing. PS: Methodology (designed and conducted field experiment) and data compilation. KS: Bacterial inoculation, disease scoring, and data analysis using spatial software. KV: Methodology (designed and conducted field experiment) and data compilation. SSR: Genotyping and conducted field experiment. MS: Genotyping (molecular analysis using SSR markers). JV: Field experiments, data tabulation, and curation. BR: Field experiments, data tabulation, and genotyping. SR: Genotyping and draft preparation. JA: Disease scoring. BM: Disease scoring. SL: Writing the original draft, and review and editing. SA: Preparing tables and figures. SK: Phenotyping for salt tolerance. All authors contributed to the article and approved the submitted version.
