# Supplementary material for: Marker-assisted enhancement of bacterial blight (Xanthomonas oryzae pv. oryzae) resistance in a salt-tolerant rice variety for sustaining rice production of tropical islands
Source: Front Plant Sci. 2023 Sep 25;14:1221537. doi: 10.3389/fpls.2023.1221537 (PMC10561094; doi:10.3389/fpls.2023.1221537)
Supplement: Supplementary file 1 [file DataSheet_1.docx]

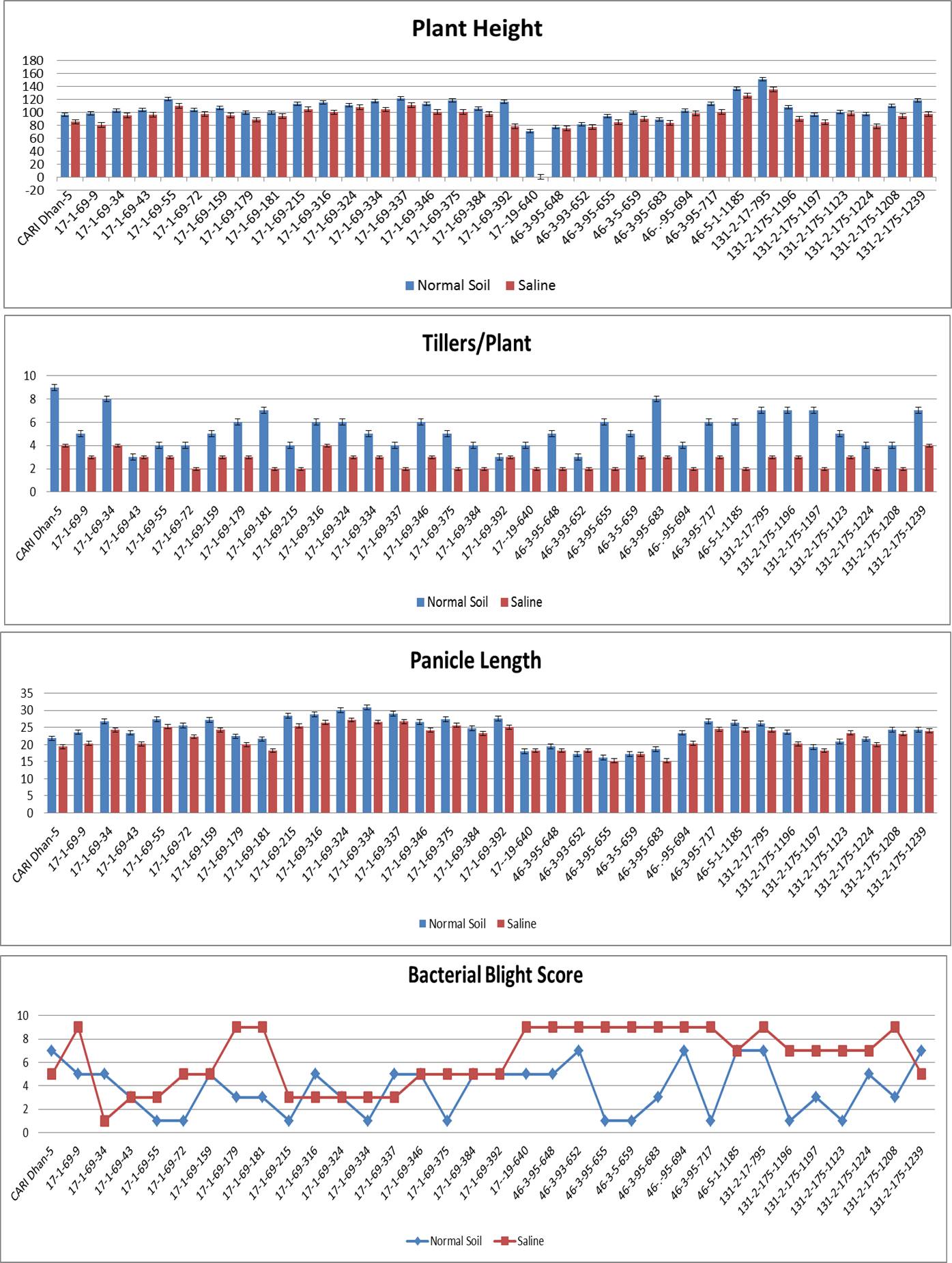


**Fig. S1** Performance of pyramided BC_3_F_1_ lines in normal and saline soil envoronment 1) Plant height, 2) Number of tillers/ plant, and 3) Panicle length

**Table S1.** Bacterial blight genes present in the progenies (CARI Dhan 5/IRBB60, BC_3_F_1_s) of CARI Dhan 5

| Sl  No. | Plant No. | BB Resistance genes | | | |
| --- | --- | --- | --- | --- | --- |
| 1 | 46-5-139 | *Xa4* | *xa5* | *xa13* | *Xa21* |
| 2 | 131-2-190 | *Xa4* | *xa5* | *xa13* | *Xa21* |
| 3 | 17-1-69 | *Xa4* | *xa5* | *xa13* | *Xa21* |
| 4 | 46-5-148 | *Xa4* | *xa5* | *xa13* | *Xa21* |
| 5 | 131-2-175 | *Xa4* | *xa5* | *xa13* | *Xa21* |
| 6 | 131-2-189 | *Xa4* | - | *xa13* | *Xa21* |
| 7 | 131-2-185 | *-* | *xa5* | *xa13* | *-* |
| 8 | 131-2-182 | - | *xa5* | *xa13* | *Xa21* |
| 9 | 17-1-51 | *Xa4* | *xa5* | *xa13* | *-* |
| 10 | 17-1-55 | *Xa4* | *xa5* | *xa13* | - |
| 11 | 17-1-49 | *-* | *xa5* | *-* | *Xa21* |
| 12 | 17-1-2 | *-* | *xa5* | *-* | *Xa21* |
| 13 | 17-1-3 | *-* | *xa5* | *-* | *Xa21* |
| 14 | 17-1-18 | *Xa4* | *xa5* | *-* | *-* |
| 15 | 46-5-143 | *Xa4* | *xa5* | *xa13* | - |
| 16 | 46-5-120 | *-* | *xa5* | *xa13* | - |
| 17 | 46-5-103 | *Xa4* | *xa5* | *-* | - |
| 18 | 46-5-107 | *Xa4* | *-* | *xa13* | - |
| 19 | 46-5-128 | *Xa4* | *xa5* | *-* | *Xa21* |
| 20 | 46-5-149 | *Xa4* | *xa5* | *-* | *Xa21* |
| 21 | 46-3-95 | *Xa4* | *xa5* | *xa13* | *Xa21* |
| 22 | 46-5-81 | *Xa4* | *-* | *-* | *Xa21* |
| 23 | 46-3-87 | *-* | *xa5* | *-* | *Xa21* |
| 24 | CARI Dhan 5 | *Xa4* | - | - | - |
| 25 | IRBB 60 | *Xa4* | *xa5* | *xa13* | *Xa21* |

**Table S2.** Performance of BILs of CARI Dhan 5 x IRBB 60 in micro-plots during Kharif 2020

| S  N | Entry No. | Days to flowering | | Plant height (cm) | | Tillers/ plant | | Panicle length (cm) | Leaf length  (cm) | | | | Leaf width  (cm) | PACP score | BB score | | |  |  |  |
| --- | --- | --- | --- | --- | --- | --- | --- | --- | --- | --- | --- | --- | --- | --- | --- | --- | --- | --- | --- | --- |
|  |  | **Normal Soil** | **Saline Soil** | **Normal Soil** | **Saline Soil** | **Normal Soil** | **Saline Soil** | **Normal Soil** | **Saline Soil** | **Normal Soil** | **Saline Soil** | **Normal Soil** | **Saline Soil** | | **Normal Soil** | **Saline Soil** | **Normal Soil** | **Saline Soil** | |  |
| 1 | IRBB 60 | 106 | 103 | 90 | 80 | 7 | 5 | 16 | 13 | 49 | 25 | 1.16 | 0.60 | | 7 | 7 | 1 | 1 | |  |
| 2 | CARI Dhan-5 | 121 | 118 | 95.8 | 85.21 | 9 | 4 | 21.8 | 19.4 | 58.6 | 30.2 | 1.32 | 0.70 | | 5 | 5 | 7 | 5 | |  |
| 3 | 17-1-69-9 | 121 | 118 | 98.6 | 80.2 | 5 | 3 | 23.6 | 20.3 | 43.6 | 30.6 | 1.46 | 0.80 | | 5 | 5 | 5 | 9 | |  |
| 4 | 17-1-69-34 | 141 | 138 | 102.8 | 95.4 | 8 | 4 | 26.8 | 24.24 | 50.6 | 51.4 | 1.12 | 1.12 | | 3 | 1 | 5 | 1 | |  |
| 5 | 17-1-69-43 | 141 | 138 | 103.4 | 96.2 | 3 | 3 | 23.4 | 20.21 | 48.4 | 44.0 | 1.14 | 1.08 | | 3 | 1 | 3 | 3 | |  |
| 6 | 17-1-69-55 | 142 | 139 | 120.4 | 110.2 | 4 | 3 | 27.4 | 25.21 | 56.4 | 40.4 | 1.36 | 0.72 | | 3 | 1 | 1 | 3 | |  |
| 7 | 17-1-69-72 | 141 | 138 | 103.6 | 97.2 | 4 | 2 | 25.6 | 22.3 | 58.2 | 42.2 | 1.36 | 1.20 | | 7 | 5 | 1 | 5 | |  |
| 8 | 17-1-69-159 | 141 | 138 | 107.2 | 95.2 | 5 | 3 | 27.2 | 24.24 | 58.2 | 43.4 | 1.36 | 0.86 | | 5 | 5 | 5 | 5 | |  |
| 9 | 17-1-69-179 | 120 | 117 | 99.4 | 88.35 | 6 | 3 | 22.4 | 20 | 57.0 | 37.8 | 1.04 | 0.96 | | 9 | 5 | 3 | 9 | |  |
| 10 | 17-1-69-181 | 120 | 117 | 99.6 | 94.5 | 7 | 2 | 21.6 | 18.2 | 36.6 | 37.0 | 1.20 | 1.00 | | 5 | 5 | 3 | 9 | |  |
| 11 | 17-1-69-215 | 141 | 138 | 113.4 | 105 | 4 | 2 | 28.4 | 25.4 | 44.8 | 48.2 | 1.34 | 1.22 | | 3 | 3 | 1 | 3 | |  |
| 12 | 17-1-69-316 | 139 | 136 | 114.8 | 100 | 6 | 4 | 28.8 | 26.4 | 48.6 | 50.8 | 1.38 | 1.30 | | 1 | 3 | 5 | 3 | |  |
| 13 | 17-1-69-324 | 141 | 138 | 111.0 | 108.2 | 6 | 3 | 30.0 | 27.2 | 60.2 | 50.2 | 1.50 | 1.08 | | 1 | 5 | 3 | 3 | |  |
| 14 | 17-1-69-334 | 126 | 123 | 117.8 | 104.2 | 5 | 3 | 30.8 | 26.58 | 61.8 | 44.2 | 1.52 | 0.94 | | 1 | 5 | 1 | 3 | |  |
| 15 | 17-1-69-337 | 128 | 125 | 122.0 | 111.2 | 4 | 2 | 29.0 | 26.7 | 58.0 | 41.8 | 1.60 | 1.08 | | 1 | 5 | 5 | 3 | |  |
| 16 | 17-1-69-346 | 142 | 139 | 113.6 | 100.5 | 6 | 3 | 26.6 | 24.2 | 60.2 | 40.8 | 1.50 | 1.04 | | 1 | 5 | 5 | 5 | |  |
| 17 | 17-1-69-375 | 126 | 123 | 118.4 | 100.8 | 5 | 2 | 27.4 | 25.6 | 56.2 | 47.2 | 1.46 | 1.06 | | 1 | 5 | 1 | 5 | |  |
| 18 | 17-1-69-384 | 126 | 123 | 105.8 | 97 | 4 | 2 | 24.8 | 23.24 | 62.4 | 46.0 | 1.34 | 1.06 | | 3 | 7 | 5 | 5 | |  |
| 19 | 17-1-69-392 | 141 | 138 | 116.6 | 78.2 | 3 | 3 | 27.6 | 25.1 | 50.0 | 44.6 | 0.96 | 0.94 | | 5 | 7 | 5 | 5 | |  |
| 20 | 46-3-95-640 | 117 | 114 | 71.0 | 75.0 | 4 | 2 | 18.0 | 18.24 | 48.4 | 32.8 | 1.14 | 1.06 | | 9 | 9 | 5 | 9 | |  |
| 21 | 46-3-95-648 | 117 | 114 | 77.4 | 75.5 | 5 | 2 | 19.4 | 18.24 | 29.6 | 41.2 | 1.28 | 0.98 | | 9 | 9 | 5 | 9 | |  |
| 22 | 46-3-93-652 | 118 | 115 | 81.2 | 77 | 3 | 2 | 17.2 | 18.2 | 41.0 | 36.0 | 1.22 | 1.02 | | 9 | 9 | 7 | 9 | |  |
| 23 | 46-3-95-655 | 122 | 119 | 94.2 | 85 | 6 | 2 | 16.2 | 15.2 | 46.4 | 42.2 | 1.26 | 0.90 | | 5 | 5 | 1 | 9 | |  |
| 24 | 46-3-95-659 | 119 | 116 | 99.2 | 90.24 | 5 | 3 | 17.2 | 17.1 | 43.6 | 31.4 | 1.52 | 0.88 | | 7 | 9 | 1 | 9 | |  |
| 25 | 46-3-95-683 | 123 | 120 | 88.6 | 83.21 | 8 | 3 | 18.6 | 15.24 | 45.2 | 38.6 | 1.04 | 1.00 | | 1 | 7 | 3 | 9 | |  |
| 26 | 46-3-95-694 | 119 | 116 | 102.4 | 98.32 | 4 | 2 | 23.4 | 20.31 | 54.6 | 47.2 | 1.46 | 1.08 | | 9 | 9 | 7 | 9 | |  |
| 27 | 46-3-95-717 | 123 | 120 | 112.8 | 100.2 | 6 | 3 | 26.8 | 24.5 | 41.4 | 44.8 | 1.28 | 1.30 | | 1 | 9 | 1 | 9 | |  |
| 28 | 46-5-149-1185 | 121 | 118 | 136.4 | 125.4 | 6 | 2 | 26.4 | 24.23 | 53.0 | 40.0 | 1.40 | 1.16 | | 7 | 7 | 7 | 7 | |  |
| 29 | 131-2-190-795 | 123 | 120 | 151.2 | 135.2 | 7 | 3 | 26.2 | 24.2 | 53.4 | 43.8 | 1.06 | 1.04 | | 1 | 9 | 7 | 9 | |  |
| 30 | 131-2-190-1196 | 119 | 116 | 107.6 | 89.5 | 7 | 3 | 23.6 | 20.2 | 63.0 | 41.4 | 0.92 | 0.74 | | 7 | 9 | 1 | 7 | |  |
| 31 | 131-2-190-1197 | 121 | 118 | 96.2 | 84.6 | 7 | 2 | 19.2 | 18.2 | 43.6 | 46.2 | 0.98 | 1.14 | | 7 | 7 | 3 | 7 | |  |
| 32 | 131-2-175-1223 | 119 | 116 | 100.8 | 98.3 | 5 | 3 | 20.8 | 23.4 | 37.2 | 44.2 | 1.08 | 1.24 | | 7 | 7 | 1 | 7 | |  |
| 33 | 131-2-175-1224 | 117 | 114 | 97.6 | 78.5 | 4 | 2 | 21.6 | 20 | 40.6 | 37.8 | 1.06 | 0.74 | | 9 | 7 | 5 | 7 | |  |
| 34 | 131-2-175-1208 | 119 | 116 | 110.4 | 94.5 | 4 | 2 | 24.4 | 23.2 | 39.6 | 38.2 | 1.22 | 0.74 | | 9 | 7 | 3 | 9 | |  |
| 35 | 131-2-175-1239 | 119 | 116 | 118.4 | 97.5 | 7 | 4 | 24.4 | 24 | 47.2 | 44.0 | 1.16 | 0.74 | | 5 | 7 | 7 | 5 | |  |
|  | **Mean** | **126.9** | **123.9** | **106.2** | **95.8** | **5.3** | **2.9** | **24.0** | **22.0** | **49.9** | **41.7** | **1.3** | **1.0** | |  |  |  |  | |  |
|  | Max | 142 | 139 | 151.2 | 135.2 | 8.75 | 4.32 | 30.8 | 27.2 | 63 | 51.4 | 1.6 | 1.3 | |  |  |  |  | |  |
|  | Min | 117 | 114 | 71 | 75.5 | 3 | 2.1 | 16.2 | 15.2 | 29.6 | 30.2 | 0.92 | 0.7 | |  |  |  |  | |  |

| **SE(m)** | 0.90 | 0.91 | 1.52 | 1.37 | 0.68 | 0.48 | 0.86 | 0.8 | 1.21 | 0.96 | 0.16 | 0.18 |
| --- | --- | --- | --- | --- | --- | --- | --- | --- | --- | --- | --- | --- |
| **SE(d)** | 10.11 | 10.11 | 15.48 | 13.49 | 1.54 | 0.78 | 4.16 | 3.71 | 8.45 | 6.10 | 0.18 | 0.18 |
| **C.V.** | 7.96 | 8.16 | 14.58 | 14.08 | 29.01 | 26.90 | 17.33 | 16.86 | 16.93 | 14.63 | 14.06 | 18.08 |

**Table S3.** Performance of BC_3_F_4_ lines in for salinity stress conditions in microplots at ICAR-CIARI, Port Blair

| **Entry No.** | **Days to flowering (50%)** | | **Plant height (cm)** | | **Panicle length (cm)** | | **BB Score** | | **Salinity tolerance score at**  **EC 5.0 dS/m^2^** | | **Grain yield/row (g)**  **(20 Plants)** | |
| --- | --- | --- | --- | --- | --- | --- | --- | --- | --- | --- | --- | --- |
|  | **EC 0.0 dS/m^2^** | **EC 5.0 dS/m^2^** | **EC 0.0 dS/m^2^** | **EC 5.0 dS/m^2^** | **EC 0.0 dS/m^2^** | **EC 5.0 dS/m^2^** | **2021** | **2022** | **2021** | **2022** | **Normal water** | **Saline stress EC 5.0 dS/m^2^** |
| **CARI Dhan-5** | 110 | 106 | 109 | 90 | 28 | 27 | 7 | 5 | 1 | 1 | 187.55 | 123.39 |
| 17-1-69-55 | 120 | 118 | 112 | 100 | 27 | 23 | 1 | 1 | 1 | 1 | 158.02 | 97.16 |
| 17-1-69-72 | 115 | 113 | 114 | 105 | 28 | 21 | 5 | 1 | 1 | 1 | 197.86 | 145.47 |
| 17-1-69-179 | 92 | 90 | 104 | 61 | 23 | 16 | 1 | 1 | 3 | 1 | 272.93 | 88.74 |
| 17-1-69-334 | 112 | 110 | 121 | 105 | 29 | 28 | 1 | 1 | 1 | 1 | 300.24 | 128.87 |
| 17-1-69-375 | 112 | 112 | 112 | 89 | 25 | 22 | 5 | 1 | 1 | 1 | 232.84 | 131.81 |
| 46-3-95-647 | 87 | 85 | 97 | 87 | 23 | 24 | 5 | 3 | 5 | 1 | 232.78 | 130.64 |
| 46-3-95-655 | 108 | 107 | 108 | 88 | 24 | 24 | 3 | 1 | 5 | 1 | 265.05 | 106.16 |
| 46-3-95-659 | 87 | 87 | 108 | 112 | 27 | 24 | 1 | 1 | 3 | 3 | 134.13 | 124.56 |
| 131-2-175-1196 | 89 | 85 | 115 | 106 | 24 | 22 | 1 | 1 | 1 | 3 | 150.51 | 49.96 |
| 131-2-175-1197 | 104 | 102 | 106 | 100 | 26 | 22 | 1 | 1 | 3 | 1 | 138.73 | 75.02 |
| 131-2-175-1209 | 85 | 86 | 98 | 102 | 23 | 20 | 3 | 3 | 3 | 3 | 180.15 | 86.35 |
| 131-2-175-1223 | 107 | 105 | 116 | 92 | 23 | 22 | 1 | 1 | 3 | 1 | 191.23 | 113.63 |
| 131-2-175-1224 | 96 | 94 | 110 | 84 | 25 | 20 | 5 | 1 | 3 | 3 | 139.38 | 93.55 |
| 131-2-175-1208 | 90 | 89 | 104 | 91 | 23 | 22 | 3 | 5 | 5 | 3 | 181.39 | 86.98 |
| 131-2-175-1239 | 105 | 103 | 104 | 90 | 26 | 21 | 3 | 3 | 3 | 3 | 209.27 | 146.88 |
| **IRBB 60** | 95 | 92 | 104 | 86 | 26 | 26 | 1 | 1 | 9 | 5 | 118 | 90 |
|  |  |  |  |  |  |  |  |  |  |  |  |  |
| **Mean** | 100.82 | 99.06 | 108.35 | 93.41 | 25.29 | 22.59 |  | | | | 193.53 | 107.01 |
| **Min** | 85 | 85 | 97 | 61 | 23 | 16 |  |  |  |  | 118 | 49.96 |
| **Max** | 120 | 118 | 121 | 112 | 29 | 28 |  |  |  |  | 300.24 | 146.88 |
| **SE(m)** | 1.13 | 1.12 | 0.61 | 1.23 | 0.4 | 0.6 |  |  |  |  | 3.9 | 2.64 |
| **SE(d)** | 11.30 | 11.13 | 6.33 | 11.85 | 2.02 | 2.85 |  |  |  |  | 52.92 | 26.63 |
| **C.V.** | 11.21 | 11.23 | 5.85 | 12.69 | 8.00 | 12.62 |  |  |  |  | 27.34 | 24.89 |

**Table S4:** Grain yield, agro-morphological and grain quality traits of parental and selected BC_3_F_4_ pyramided lines in replicated yield trials (normal soil).

| **Treatment** | **Days to flowering (50%)** | **Days to maturity** | **Plant height (cm)** | **Effective tillers/plant** | **Panicle length (cm)** | **Grain length (mm)** | **Grain width (mm)** | **Grain L:W Ratio** | **1000 grain weight (g)** | **Grain yield (Kg/ha)** | **Yield advantage over the recurrent parent (%)** |
| --- | --- | --- | --- | --- | --- | --- | --- | --- | --- | --- | --- |
| CARI Dhan 5 | 108 | 138 | 129 | 7.00 | 25.67 | 8.64 | 2.96 | 2.92 | 28.23 | 4434.16 | 00.00 |
| 17-1-69-55 | 123 | 153 | 120 | 6.33 | 25.00 | 8.86 | 2.96 | 2.99 | 28.80 | 4115.23 | -07.19 |
| 17-1-69-72 | 81 | 111 | 123 | 6.00 | 26.67 | 8.60 | 2.82 | 3.05 | 28.17 | 3868.31 | -12.76 |
| 17-1-69-179 | 90 | 120 | 110 | 5.33 | 23.00 | 8.64 | 2.86 | 3.02 | 27.33 | 3261.32 | -26.45 |
| 17-1-69-334 | 111 | 141 | 112 | 6.00 | 25.67 | 8.92 | 2.93 | 3.04 | 26.80 | 3004.12 | -32.25 |
| 17-1-69-375 | 115 | 145 | 118 | 6.00 | 26.33 | 8.85 | 2.79 | 3.17 | 28.70 | 5838.48 | 31.67 |
| 17-1-69-647 | 87 | 117 | 105 | 6.00 | 24.33 | 8.56 | 2.77 | 3.10 | 25.93 | 5997.94 | 35.27 |
| 17-1-69-655 | 106 | 136 | 116 | 5.67 | 26.00 | 8.80 | 2.80 | 3.15 | 26.93 | 4063.79 | -08.35 |
| 17-1-69-659 | 87 | 117 | 112 | 6.33 | 23.00 | 8.50 | 2.82 | 3.01 | 25.70 | 6183.13 | 39.44 |
| 17-2-190-1196 | 87 | 117 | 129 | 6.00 | 22.67 | 8.26 | 2.69 | 3.07 | 24.57 | 3827.16 | -13.69 |
| 17-2-190-1197 | 104 | 134 | 113 | 6.33 | 22.67 | 8.69 | 2.98 | 2.92 | 28.53 | 3909.47 | -11.83 |
| 17-2-175-1209 | 88 | 118 | 105 | 5.67 | 22.67 | 8.61 | 2.64 | 3.26 | 23.13 | 4629.63 | 04.41 |
| 17-2-175-1223 | 107 | 137 | 119 | 5.67 | 23.67 | 8.31 | 2.53 | 3.28 | 21.60 | 4526.75 | 02.09 |
| 17-2-175-1224 | 94 | 124 | 111 | 6.00 | 21.33 | 8.35 | 2.65 | 3.15 | 21.73 | 3271.61 | -26.22 |
| 17-2-175-1208 | 89 | 119 | 109 | 5.67 | 23.33 | 8.48 | 2.57 | 3.31 | 22.07 | 3858.03 | -12.99 |
| 17-2-175-1239 | 105 | 135 | 121 | 6.67 | 24.67 | 8.63 | 2.69 | 3.22 | 23.40 | 4197.53 | -05.34 |
| **Mean** | **98.88** | **128.88** | **115.73** | **6.04** | **24.04** | **8.69** | **2.76** | **3.16** | **25.88** | **4300.11** |  |
| **C.D.** | **23.57** | **23.57** | **10.79** | **N/A** | **1.86** | **0.30** | **0.12** | **0.19** | **1.10** | **800.53** |  |
| **SE(m)** | **8.14** | **8.14** | **3.73** | **0.42** | **0.64** | **0.11** | **0.04** | **0.07** | **0.38** | **276.64** |  |
| **SE(d)** | **11.52** | **11.52** | **5.27** | **0.60** | **0.91** | **0.15** | **0.06** | **0.09** | **0.54** | **391.23** |  |
| **C.V.** | **14.27** | **10.95** | **5.58** | **12.08** | **4.62** | **2.09** | **2.66** | **3.57** | **2.55** | **11.14** |  |

**Table S5:** Performance of CARI Dhan 5 BILs under sodic soil microplots in replicated yield trials.

| **Entry No./ Traits observed** | **Sodic Score** | | **Yield/ plant**  **(20 plants)** | | **Plant height** | | **Panicle length** | | **Productive tillers/ plant** | |
| --- | --- | --- | --- | --- | --- | --- | --- | --- | --- | --- |
|  | **High (pH 9.9)** | **Medium**  **(pH 9.5)** | **High** | **Medium** | **High** | **Medium** | **High** | **Medium** | **High** | **Medium** |
| **CARI Dhan 5** | 7 | 5 | 119.0 | 137.2 | 66.5 | 79.5 | 19.5 | 22 | 7.5 | 8 |
| 17-1-69-55 | 9 | 3 | - | 141.0 | - | 71 | - | 23 | - | 5 |
| 17-1-69-72 | 7 | 3 | 53.7 | 171.0 | 53.5 | 75.5 | 17.5 | 22.5 | 3.5 | 5 |
| 17-1-69-179 | 7 | 3 | 81.0 | 80.1 | 55 | 68.5 | 17.5 | 18.5 | 3 | 5 |
| 17-1-69-334 | 9 | 3 | - | 72.9 | - | 73.5 | - | 23 | - | 4 |
| 17-1-69-375 | 9 | 3 | - | 126.5 | - | 75 | - | 23 | - | 5.5 |
| 46-3-95-647 | 3 | 3 | 124.0 | 160.0 | 54.5 | 73 | 18.5 | 25 | 4.5 | 4.5 |
| 46-3-95-655 | 5 | 5 | 82.0 | 90.7 | 62.5 | 82 | 19 | 23 | 4 | 5.5 |
| 46-3-95-659 | - | - | - | - | - | - | - | - | - | - |
| 131-2-175-1196 | 3 | 3 | 131.3 | 153.0 | 56 | 91.5 | 18.5 | 23 | 3.5 | 4 |
| 131-2-175-1197 | 5 | 3 | 76.7 | 95.0 | 55 | 70.5 | 18.5 | 22 | 4 | 4 |
| 131-2-175-1209 | 3 | 3 | 100.0 | 138.5 | 56.5 | 81.5 | 18 | 26.5 | 6 | 7 |
| 131-2-175-1223 | 7 | 3 | 67.7 | 88.0 | 49 | 72.5 | 14.5 | 21.5 | 4.5 | 5.5 |
| 131-2-175-1224 | 3 | 3 | 124.7 | 147.9 | 68 | 105.5 | 16.5 | 28.5 | 4.5 | 4.5 |
| 131-2-175-1208 | 5 | 3 | 94.3 | 130.0 | 55 | 78.5 | 18 | 21 | 5.5 | 6.5 |
| 131-2-175-1239 | 7 | 5 | 80.0 | 117.0 | 56.5 | 73 | 18 | 24 | 6 | 7 |
| **IRBB 60** | 7 | 7 | 71.3 | 81.0 | 59 | 66 | 17.5 | 18 | 4 | 4 |
| **Mean** |  |  | 92.75 | 120.61 | 57.46 | 77.31 | 17.81 | 22.78 | 4.65 | 5.31 |
| **Min** |  |  | 53.7 | 72.9 | 49 | 66 | 14.5 | 18 | 3 | 4 |
| **Max** |  |  | 131.3 | 171 | 68 | 105.5 | 19.5 | 28.5 | 7.5 | 8 |
| **SE(m)** |  |  | 2.67 | 2.97 | 0.7 | 1.11 | 0.3 | 0.55 | 0.6 | 0.55 |
| **SE(d)** |  |  | 25.02 | 31.76 | 5.31 | 9.72 | 1.25 | 2.59 | 1.26 | 1.24 |

**Table S6:** Distinctness, Uniformity and Stability (DUS) characterization of BC_3_F_4_ lines under field conditions

| **Characteristics** | **States** | **Note** | **CARI Dhan 5** | **17-1-69-55** | **17-1-69-72** | **17-1-69-179** | **17-1-69-334** | **17-1-69-375** | **46-3-95-647** | **46-3-95-655** | **46-3-95-659** | **131-2-190-1196** | **131-2-190-1197** | **131-2-175-1209** | **131-2-175-1223** | **131-2-175-1224** | **131-2-175-1208** | **131-2-175-1239** |
| --- | --- | --- | --- | --- | --- | --- | --- | --- | --- | --- | --- | --- | --- | --- | --- | --- | --- | --- |
| Coleoptile: Colour | Colourless | 1 | 1 | 1 | 1 | 1 | 1 | 1 | 1 | 1 | 1 | 1 | 1 | 1 | 1 | 1 | 1 | 1 |
| Basal leaf: Sheath colour | Green | 1 | 1 | 1 | 1 | 1 | 1 | 1 | 1 | 1 | 1 | 1 | 1 | 1 | 1 | 1 | 1 | 1 |
| Leaf: intensity of green colour | Medium | 5 | 5 | 5 | 5 | 5 | 5 | 5 | 5 | 5 | 5 | 5 | 5 | 5 | 5 | 5 | 5 | 5 |
| Leaf: pubescence of blade surface | Strong | 7 | 7 | 7 | 7 | 7 | 7 | 7 | 7 | 7 | 7 | 7 | 7 | 7 | 7 | 7 | 7 | 7 |
| Leaf: auricles | Present | 9 | 9 | 9 | 9 | 9 | 9 | 9 | 9 | 9 | 9 | 9 | 9 | 9 | 9 | 9 | 9 | 9 |
| Leaf: anthocyanin colouration of auricles | Colourless | 1 | 1 | 1 | 1 | 1 | 1 | 1 | 1 | 1 | 1 | 1 | 1 | 1 | 1 | 1 | 1 | 1 |
| Leaf: collar | Present | 9 | 9 | 9 | 9 | 9 | 9 | 9 | 9 | 9 | 9 | 9 | 9 | 9 | 9 | 9 | 9 | 9 |
| Leaf: anthocyanin colouration of collar | Absent | 1 | 1 | 1 | 1 | 1 | 1 | 1 | 1 | 1 | 1 | 1 | 1 | 1 | 1 | 1 | 1 | 1 |
| Leaf: ligule | Present | 9 | 9 | 9 | 9 | 9 | 9 | 9 | 9 | 9 | 9 | 9 | 9 | 9 | 9 | 9 | 9 | 9 |
| Leaf: shape of ligule | Truncate | 3 | 3 | 3 | 3 | 3 | 3 | 3 | 3 | 3 | 3 | 3 | 3 | 3 | 3 | 3 | 3 | 3 |
| Leaf: colour of ligule | White | 1 | 1 | 1 | 1 | 1 | 1 | 1 | 1 | 1 | 1 | 1 | 1 | 1 | 1 | 1 | 1 | 1 |
| Leaf: length of blade | Med.(30-35cm) | 5 |  |  |  |  |  |  |  |  |  |  |  | 5 |  |  |  |  |
|  | Long(>45cm) | 7 | 7 | 7 | 7 | 7 | 7 | 7 | 7 | 7 | 7 | 7 | 7 |  | 7 | 7 | 7 | 7 |
| Leaf: width of blade | Med.(1-2cm) | 5 | 5 |  | 5 | 5 | 5 |  | 5 | 5 | 5 |  | 5 | 5 | 5 | 5 | 5 | 5 |
|  | Broad(>2cm) | 7 |  | 7 |  |  |  | 7 |  |  |  | 7 |  |  |  |  |  |  |
| Time of heading (50% of plants with panicles) (days) | Late (111-130) | 7 | 7 | 7 | 7 | 7 | 7 | 7 | 7 | 7 | 7 | 7 | 7 | 7 | 7 | 7 | 7 | 7 |
| Flag leaf: attitude of blade (early observation) | Erect | 1 | 1 | 1 | 1 | 1 | 1 | 1 | 1 | 1 | 1 | 1 | 1 | 1 | 1 | 1 | 1 | 1 |
| Spikelet: density of pubescence of lemma | Medium | 5 | 5 | 5 | 5 | 5 | 5 | 5 | 5 | 5 | 5 | 5 | 5 | 5 | 5 | 5 | 5 | 5 |
| Lemma: anthocyanin colouration of area below keel | Absent | 1 | 1 | 1 | 1 | 1 | 1 | 1 | 1 | 1 | 1 | 1 | 1 | 1 | 1 | 1 | 1 | 1 |
| Lemma: anthocyanin colouration of area below apex | Absent | 1 | 1 | 1 | 1 | 1 | 1 | 1 | 1 | 1 | 1 | 1 | 1 | 1 | 1 | 1 | 1 | 1 |
| Lemma: anthocyanin colouration of area below apex | Absent | 1 | 1 | 1 | 1 | 1 | 1 | 1 | 1 | 1 | 1 | 1 | 1 | 1 | 1 | 1 | 1 | 1 |
| Spikelet: colour of stigma | White | 1 | 1 | 1 | 1 | 1 | 1 | 1 | 1 | 1 | 1 | 1 | 1 | 1 | 1 | 1 | 1 | 1 |
| Stem: thickness | Thin (<0-40cm) | 3 |  | 3 |  |  |  |  |  | 3 | 3 | 3 | 3 | 3 |  |  |  |  |
|  | Medium (0.40-0.55cm) | 5 | 5 |  | 5 | 5 | 5 | 5 | 5 |  |  |  |  |  |  |  |  |  |
| Stem: length (excluding panicle; excluding floating rice) | Short(91-110cm) | 3 | 3 | 3 | 3 | 3 | 3 | 3 | 3 | 3 | 3 | 3 | 3 | 3 | 3 | 3 | 3 | 3 |
| Stem: anthocyanin colouration of nodes | Absent | 1 | 1 | 1 | 1 | 1 | 1 | 1 | 1 | 1 | 1 | 1 | 1 | 1 | 1 | 1 | 1 | 1 |
|  | Present | 9 |  |  |  |  |  |  |  |  |  |  |  |  |  |  |  |  |
| Panicle: length of main axis | Medium(21-25cm) | 5 |  |  |  |  |  |  |  | 5 | 5 |  |  | 5 | 5 | 5 | 5 | 5 |
|  | Long(26-30cm) | 7 | 7 | 7 | 7 | 7 |  |  | 7 |  |  | 7 | 7 |  |  |  |  |  |
|  | Very long(>30cm) | 9 |  |  |  |  | 9 | 9 |  |  |  |  |  |  |  |  |  |  |
| Flag leaf: attitude of blade (late observation) | Erect | 1 | 1 | 1 | 1 | 1 | 1 | 1 | 1 | 1 | 1 | 1 | 1 | 1 | 1 | 1 | 1 | 1 |
| Panicle: curvature of main axis | Straight | 1 | 7 | 7 | 7 | 7 | 7 | 7 | 7 | 7 | 7 | 7 | 7 | 7 | 7 | 7 | 7 | 7 |
| Panicle: number per plant | Few (<11) | 3 | 3 | 3 | 3 | 3 | 3 | 3 | 3 | 3 | 3 | 3 | 3 | 3 | 3 | 3 | 3 | 3 |
| Spikelet: colour of tip of lemma | Brown | 3 | 3 | 3 | 3 | 3 | 3 | 3 | 3 | 3 | 3 | 3 | 3 | 3 | 3 | 3 | 3 | 3 |
| Lemma and Palea: colour | Gold and gold | 2 | 2 | 2 | 2 | 2 | 2 | 2 | 2 | 2 | 2 | 2 | 2 | 2 | 2 | 2 | 2 | 2 |
| Panicle: awns | Absent | 1 | 1 | 1 | 1 | 1 | 1 | 1 | 1 | 1 | 1 | 1 | 1 | 1 | 1 | 1 | 1 | 1 |
| Panicle: exsertion | Partly exserted | 3 | 3 | 3 | 3 | 3 | 3 | 3 | 3 | 3 | 3 | 3 | 3 | 3 | 3 | 3 | 3 | 3 |
|  | Late (141-160) | 7 | 7 | 7 | 7 | 7 | 7 | 7 | 7 | 7 | 7 | 7 | 7 | 7 | 7 | 7 | 7 | 7 |
| Leaf: senescence | Late | 7 | 7 | 7 | 7 | 7 | 7 | 7 | 7 | 7 | 7 | 7 | 7 | 7 | 7 | 7 | 7 | 7 |
| Sterile lemma: colour | Straw | 1 | 1 | 1 | 1 | 1 | 1 | 1 | 1 | 1 | 1 | 1 | 1 | 1 | 1 | 1 | 1 | 1 |
| Grain: weight of 1000 fully developed grains | Medium (21-25g) | 5 | 5 | 5 | 5 | 5 | 5 | 5 | 5 | 5 | 5 | 5 | 5 | 5 | 5 | 5 | 5 | 5 |
| Grain length | Medium (8.6-10.5mm) | 5 | 5 | 5 | 5 | 5 | 5 | 5 | 5 | 5 | 5 | 5 | 5 | 5 | 5 | 5 | 5 | 5 |
| Grain width | Narrow (2.1-2.5mm) | 3 |  |  |  |  |  |  |  |  |  |  |  |  |  | 3 |  |  |
|  | Medium (2.6-3.0mm) | 5 | 5 | 5 | 5 | 5 | 5 | 5 | 5 | 5 | 5 | 5 | 5 | 5 | 5 |  | 5 | 5 |
| Decorticated grain length | Medium | 3 | 3 | 3 | 3 | 3 | 3 | 3 | 3 | 3 | 3 | 3 | 3 | 3 | 3 | 3 | 3 |  |
|  | Long | 5 |  |  |  |  |  |  |  |  |  |  |  |  |  |  |  | 5 |
| Decorticated grain width | Medium (2.0-2.5mm) | 5 |  | 5 |  |  | 5 | 5 | 5 | 5 | 5 | 5 |  | 5 | 5 | 5 | 5 |  |
|  | Broad(>2.5mm) | 7 | 7 |  | 7 | 7 |  |  |  |  |  |  | 7 |  |  |  |  | 7 |
| Decorticated grain shape (in lateral view) | Short slender | 1 |  |  |  |  |  |  |  |  |  |  |  |  |  |  |  |  |
|  | Short bold | 2 |  |  |  |  |  |  |  |  |  |  |  |  |  |  |  |  |
|  | Medium slender | 3 |  |  |  |  |  |  |  |  |  |  |  |  |  |  |  |  |
|  | Long bold | 4 | 4 | 4 | 4 | 4 | 4 | 4 | 4 | 4 | 4 | 4 | 4 | 4 | 4 | 4 | 4 |  |
|  | Long slender | 5 |  |  |  |  |  |  |  |  |  |  |  |  |  |  |  | 5 |
|  | Long slender* (for basmati type) Extra-long slender | 6 |  |  |  |  |  |  |  |  |  |  |  |  |  |  |  |  |
| Decorticated grain colour | White | 1 | 1 | 1 | 1 | 1 | 1 | 1 | 1 | 1 | 1 | 1 | 1 | 1 | 1 | 1 | 1 | 1 |
| Decorticated grain aroma | Absent | 1 | 1 | 1 | 1 | 1 | 1 | 1 | 1 | 1 | 1 | 1 | 1 | 1 | 1 | 1 | 1 | 1 |
